# Anti-Müllerian hormone, sex steroids, and metabolic profile in cord blood of pregnancies with type 2 diabetes and gestational diabetes

**DOI:** 10.3389/fendo.2025.1589541

**Published:** 2025-07-28

**Authors:** Claudio Villarroel, Patricia López, Soledad Henriquez, Paulina Kohen, Ethel Codner

**Affiliations:** ^1^ Institute for Maternal and Child Research (IDIMI), School of Medicine, University of Chile, Santiago, Chile; ^2^ Hospital Clínico San Borja Arriarán, Servicio de Salud Centro, Ministerio de Salud, Santiago, Chile

**Keywords:** diabetes in pregnancy, ovary, insulin resistance, anti-Müllerian hormone, androgens, fetal programming

## Abstract

**Context:**

The prevalence of type 2 diabetes (T2D) and gestational diabetes (GD)among women of reproductive age has increased in recent decades, making it the most common pregnancy complication. Many studies have examined pregnancy complications in women with diabetes; however, the impact of diabetes on the intrauterine environment, specifically ovarian markers and metabolic profiles in very preterm infants at birth, has not been studied. This study aimed to investigate AMH, sex steroid levels, and the metabolic profile in venous cord blood (VCB) in gestations affected by type 2 diabetes (T2D) and gestational diabetes (GD).

**Material and methods:**

Hormonal profile was evaluated in VCB of pregnancies with T2D (n=24), GD (n=26), and pregnancies without diabetes (C, n=25). Only pregnancies carrying a female offspring were included. AMH, sex steroids, and metabolic function biomarkers, including glucose, insulin, IGF-1, and adiponectin (APN) were measured. Clinical and anthropometric data were assessed in the mothers and offspring.

**Results:**

AMH VCB levels were significantly higher in T2D than in GD and C pregnancies (P<0.01 and P<0.005, respectively). Dehydroepiandrosterone sulfate (DHEAS) and sex hormone-binding globulin (SHBG) VCB levels were lower in T2D pregnancies than in GD and C (P < 0.01, P < 0.0001, respectively). APN levels were lower in T2D pregnancies than in C (P < 0.05). Additionally, higher insulin and IGF-1 VCB levels and HOMA-IR index were observed in T2D than in C and GD (P < 0.001, P<0.05, and P<0.05, respectively). No significant correlations were observed between maternal and AMH, insulin, IGF-1, and androgen VCB levels.

**Discussion:**

T2D disrupts the intrauterine environment, leading to increased insulin, IGF-1, HOMA-IR, and AMH concentrations and decreased adiponectin levels in VCB. These findings describe the impact that maternal T2D may have on the health and development of their offspring.

## Introduction

An increase in the prevalence of type 2 diabetes (T2D) among women of reproductive age has been observed in recent decades. Concomitantly, the incidence of gestational diabetes (GD) has increased by 10% in the last twenty years, becoming the most common complication of pregnancy (1, 2).

Despite the numerous studies that have evaluated the complications of pregnancy and delivery in women with diabetes (3, 4), there is no information about how maternal diabetes could affect the ovarian hormones at birth. Anti-Müllerian hormone (AMH) is an essential glycoprotein secreted by granulosa cells of the preantral and small antral follicles. AMH regulates ovarian development and follicle growth from the primordial to the primary follicle stage in intrauterine and postnatal life (5, 6). However, AMH levels have not been evaluated in venous cord blood (VCB) samples of pregnant women with diabetes.

Adiponectin (APN), one of the most abundant adipokines, is secreted by the placenta, vascular cells, and fetal adipocytes (7–9). Studies in newborns have shown that lower APN levels are associated with higher adipose tissue deposition and increased insulin resistance (9). Previously, lower APN levels have been described in venous cord blood and neonates of pregnancies with type 1 diabetes (T1D) (7, 10, 11). A role of APN in the relationship of fetal and placental growth has been described in these pregnancies with T1D (7, 10). However, there is scarce information about the APN levels in VCB of pregnancies with T2D and their association with insulin sensitivity, fetal growth, and ovarian hormones.

The present study aimed to investigate the impact of T2D and GD on the intrauterine environment by measuring AMH, sex steroids, APN, insulin, and IGF-1 levels in the VCB of pregnancies with T2D and GD, compared to hormonal profile in VCB of pregnancies with normal glucose levels and to analyze the hormonal profiles of the corresponding mothers at the time of delivery. We hypothesize that maternal hyperglycemia adversely disrupts markers of ovarian function, APN levels, and metabolic profile in VCB. We postulate that the hormonal profile changes in VCB of T2D pregnancies are more severe than in those with GD, due to the earlier and greater metabolic disruption observed in the former group.

## Materials and methods

### Study design and participants

A prospective study of mothers and their female offspring (n=75) was conducted during the second half of pregnancy and delivery. In the present study, we report AMH, sex hormone levels, and metabolic profiles in samples obtained in VCB of pregnancies with T2D (T2D, n = 24) and GD (GD; n =26), compared to a control group of pregnancies without diabetes (C, n=25). The maternal clinical and hormonal profiles during the second and third trimesters of pregnancy and the study design have previously been reported (12, 13). Briefly, mothers with T2D (T2D, n=24) and mothers with gestational diabetes (GD, n=26) were recruited from the Fetal–Maternal Unit of the Hospital Clínico San Borja Arriarán, a tertiary hospital; the mothers in the control group (C, n=25) were recruited from nearby local clinics. T2D were diagnosed before pregnancy according to the WHO definition (14). GD was defined according to the following criteria: a normal fasting glucose level during the first trimester of gestation (< 100 mg/dl) and a fasting glucose level ≥ 100 mg/dl and/or a 2-hour glucose level on a 75-g oral glucose tolerance test (OGTT) ≥ 140 mg/dl at 24-28 weeks (14). The control group had normal first-trimester fasting glucose and 75-g OGTT results at 24-28 weeks of gestation (14). The glucose and HbA1c levels during pregnancy were previously reported (12, 13).

Only women with spontaneous singleton pregnancies with a female fetus were included. The exclusion criteria were as follows: preterm delivery before 34 weeks of gestation, multiple pregnancy, male fetus, fetus with severe malformations, diagnosis of polycystic ovary syndrome (PCOS), the presence of hyperandrogenism from other causes, use of corticoids or steroids, use of ovulation induction drugs, pregnancy by *in vitro* fertilization, or the presence of a severe chronic disease.

The Institutional Review Board of San Borja Arriarán Hospital approved the protocol, and all the mothers signed the informed consent form and agreed to the study of their offspring.

### Study protocol

A clinical assessment of the pregnant mother was performed at the time of delivery, as previously described (12, 13). Maternal venous blood samples were obtained from T2D, GD, and C at admission to the hospital for delivery to measure AMH, sex steroids, APN, and metabolic parameters, including glucose, insulin, HOMA-IR index, IGF-1, IGFBP-1 in maternal plasma, and HbA1 was determined in whole blood. A clinical assessment was conducted on the female newborns, and VCB samples were collected.

Clinical assessment of the offspring was performed at birth, including weight, length, head circumference, and gestational age. Ponderal index and standard deviation scores for birth weight (birth weight SDS) and birth length (birth length SDS) were calculated using Chilean standards (15). Newborns were classified as small for gestational age (SGA) if their birth weight was at or below the 10th percentile, adequate for gestational age (AGA) if birthweight was more than 10th and less than 90th percentiles, and large for gestational age (LGA) if at or above the 90th percentile (16).

A VCB sample (20 ml) was obtained from the umbilical vein after clamping and collected into a container with lithium heparin at room temperature to assess the AMH, sex steroids, APN and metabolic profiles in plasma obtained from VCB. The sample was then immediately centrifuged, and the resulting plasma was stored at -20°C.

### Hormonal measurements

The following methods were used for the assessment of hormonal measurements in maternal and VCB plasma: AMH was quantified with Gen II ELISA kits (AMH, cat A79765, Beckman Coulter, Inc., USA) (12, 13), with an assay sensitivity (S) of 0.08 ng/ml and intra- and inter-assay coefficient of variation (CV) of 5.3 and 8.7%, respectively. Five cases showed AMH levels at the lower threshold of 0.08 ng/mL, in these cases, a value of 0.08 ng/ml was added to the database for statistical analysis.

SHBG levels were measured by immunoradiometric assay (IRMA) specific kit (SHBG, catalog # L2SH12, RRID: AB_2750986), Siemens Healthcare Diagnostics) with S of 0.5 nmol/l; intra-assay CV and inter-assay CV were 3.9% and 6.9%, respectively. Total testosterone (S = 0.01 ng/ml), androstenedione (S = 0.02 ng/ml), and DHEAS (S = 25.0 ng/l) were measured by radioimmunoassay specific kits (RIA, testosterone catalog # DSL-4000, RRID: AB_3096130, androstenedione catalog# ab178609, RRID: AB_2811019, DHEAS catalog # DSL-8900, RRID: AB_3096133 respectively, DIAsource ImmunoAssays SA). Intra-assay CVs were 4.2%, 7.7%, and 4.1% for testosterone, androstenedione and DHEAS, respectively. Inter-assay CVs were 8.9%, 7.3%, and 6.1% for testosterone, androstenedione and DHEAS, respectively. Free androgen index was calculated as previously reported (12, 13).

Estradiol (E2, S = 0.06 ng/l), estrone (E1, S = 0.03 ng/l), and estriol (E3, S = 0.075 ng/ml) were measured by specific RIA kits (E2, Catalog # 10490889, RRID: AB_2895133; E1, Catalog # E3135, RRID: AB_259293) and E3, Catalog # LS-C56446-100, RRID: AB_1509903); respectively; Siemens Healthcare Diagnostics). Intra-assay coefficients of variation (CV) were 6.1%, 6.2%, and 3.6% for E2, E1, and E3, respectively. Inter-assay CVs were 8.6%, 12.2%, and 7.5% for E2, E1, and E3, respectively (12, 13).

Glucose was measured using the hexokinase-glucose-6-phosphate dehydrogenase method. The intra-assay and inter-assay CVs were 1.5% and 1.3%, respectively. HbA1c was measured using a state-of-the-art commercially available automatic system (Siemens DCA System). Insulin (S = 1 μIU/ml) was measured by IRMA specific kit (Insulin, catalog# LS-C85236-100, RRID: AB_1665399, DIAsource ImmunoAssays SA). Intra-assay and inter-assay CVs were 1.5% and 6.1%, respectively. The homeostatic model assessment of insulin resistance (HOMA-IR) was calculated as previously reported (12, 13).

IGF-1 (S = 3.4 ng/ml) and IGFBP-1 (S = 0.1 ng/ml) were measured by RIA and ELISA, respectively (IGF-1, catalog # 5217-1, RRID: AB_10895981; IGFBP-1; catalog # MA5-23727, RRID: AB_2609599, respectively, DIAsource Immunoassays SA). Intra-assay CVs were 4.2% for IGF-1 and 6.8% for IGFBP-1. Inter-assay CVs were 6.5% for IGF-1 and 7.4% for IGFBP-1.

Adiponectin (S = 0.6 ng/ml) was measured by ELISA (adiponectin, catalog # MAB3667, RRID: AB_10548434, DIAsource ImmunoAssays S.A., Louvain-La-Neuve, Belgium). The intra- and inter-assay CVs were 4.7% and 6.7%, respectively.

### Statistical analysis

The normality of the variables was checked with the Kolmogorov–Smirnov test. However, the clinical and hormonal parameters did not meet the normality criteria. Therefore, nonparametric statistics were used to analyze the data. The results are presented as the median value along with the minimum and maximum range.

Continuous variables were analyzed with the Kruskal–Wallis Test with pairwise comparisons that were adjusted by the Bonferroni correction for multiple tests. Comparisons of the prevalence of SGA, and LGA among the three groups of newborns were performed with Pearson Chi-square test. A paired sample correlation analysis between maternal plasma and VCB hormonal levels was done using Pearson’s two-tailed test.

The sample size calculation has been previously reported (12, 13). All the statistical calculations were conducted with SPSS Statistics 21 (IBM), and P < 0.05 indicated statistical significance.

## Results

### Clinical characteristics of the newborn female offspring

The gestational ages of the daughters of T2D were lower than the C group (**Table 1**; P < 0.005). The daughters of the T2D group had a higher birth weight SDS than the GD (P <0.05), and the offspring of the T2D and GD groups also had a higher ponderal index and LGA prevalence than the C group (P <0.005 and P <0.05, respectively). The proportion of SGA infants and the remaining anthropometric measurements were similar among the three groups.

**Table 1:** Clinical characteristics at the birth of the female offspring of pregnant mothers with T2D, GD, or in the control (C) group. Data are shown as median (minimum-maximum).

Offspring			
	Daughter T2D	Daughter GD	Daughter control
n	24	26	25
Gestational Age (weeks)	38 (34-40.3)^a^	38.0 (34-41)	39 (36-40)
Birth Weight (g)	3580 (2320-4770)	3230 (2480-4770)	3440 (2260-4320)
Birth Weight SDS	1.0 (-0.8 -4.0) ^b^	-0.3 (-1.8-2.4)	0.5 (-2.1-2.0)
Length (cm)	47 (40-53)	46.7 (42.0-50)	49.5 (41-52)
Length SDS	-0.1 (-6.9-1.4)	-0.5 (-3.5-1.4)	-0.1 (-6.9-1.0)
Head Circumference (cm)	34 (32-38)	34 (31-36)	34 (32-36.5)
Ponderal Index (g/cm^3^)	3.4 (3.0 - 3.7)^a^	3.5 (3.1-4.3)^c^	2.9 (2.1–5.5)
Large for Gestational Age (n, %)	12 (50)	9 (34.6)	4 (16.0)*
Small for Gestational Age (n, %)	0 (0)	3 (11.5)	2 (8.0)

Mothers			
	Mother T2D	Mother GD	Mother control
n	24	26	25
Age (years)	32.9 (25.9-43)^a^	32.3 (20-43)	29.7 (17.3-42.1)
BMI (kg/m^2^)	37.8 (25.0-47.3)^d^	34.7 (23.3-48.5)^e^	31.2 (27.3-41.1)
Diet only	4(16.7)	10 (38.5)	N/A
Metformin only (n, %)	2 (8.3)^+^	9 (34.6)	N/A
Insulin only (n, %)	17 (70.8)^++^	5 (19.2)	N/A
Metformin + insulin (n, %)	1(4.2)	2 (7.6)	N/A
HbA1c (%)	6.2 (4.9-10.7)^d^	5.4 (4.7-6.3)^c^	5.1 (4.2-5.7)
HOMA-IR	4.7 (1.2-17.8)^d^	1.8 (0.4-9.1)^c^	2.3 (0.6-5.5)

Data are presented as medians (minimum to maximum). Symbols: In the upper part of **Table 1** (offspring data): ^a^P< 0.005 for T2D vs. C, and ^b^P< 0.05 for T2D vs. GD. ^c^: P<0.0001 GD vs C; ^d^:P<0.0001 T2D vs C; ^e^: P<0.05 GD vs C. *: P<0.05 Proportion in the three groups.

N/A, Not applicable. +: P<0.05 T2D vs GD. ++ p<0.005 T2D vs GD.

The individuals in the control group, as defined in the exclusion criteria, were not treated with any medications and had a regular diet.

### Clinical characteristics, androgen levels, and metabolic profiles of the mothers

In the maternal group, the T2D were older than the C group (**Table 1**, P< 0.005). BMI was higher in the mothers with T2D and GD compared to C groups (P< 0.0001). A higher proportion of mothers with T2D received insulin or metformin treatment than those with GD (P<0.005 and P <0.05, respectively).

HbA1c levels and HOMA-IR index were higher in mothers with T2D and GD compared to C (P<0.0001).

### Plasma sex steroid and AMH levels in VCB samples

AMH VCB levels were significantly elevated in the T2D compared to GD and C (**Table 2**, P<0.01 and P<0.005 respectively). These differences in AMH levels remained significant after excluding the five cases with AMH below the detection level from the analysis.

**Table 2:** Plasma sex steroid and AMH levels of venous cord blood of T2D, GD, and control pregnancies.

Venous blood cord levels	T2D	GD	Control
n	24	26	25
AMH (ng/ml)	1.5 (0.1-4.6)^a,b^	0.7 (0.1-1.2)	0.2 (0.1-1.2)
SHBG (nmol/l)	166.1 (132-243) ^c,d^	199.1 (173-312)	187 (81-232)
Testosterone (ng/ml)	1.7 (0.7-3.65)	1.2 (0.7-6.9)	1.8 (0.2-3.6)
Androstenedione (ng/ml)	26.3 (16.1 - 40.0)	22.4 (14.2-40)	26.8 (5.6-38)
DHEAS (μg/dl)	149.9 (98.3-243.8)^b,e^	198.7 (75.5-702.6)	216.7 (113.6-366.7)
Free Androgen Index (%)	3.5 (1.4–7.1)	2.1 (1.4-7.6)	3.4 (0.3-6.4)
Estradiol (ng/ml)	10.3 (7.4-23.9)	10.8 (4.1-145.5)	9.4 (4.7-26.1)
Estrone (ng/ml)	7754 (3274 - 21832)	12744 (3364-34688)	11443 (2101-36014)
Estriol (ng/ml)	64.3 (32-109.2)	58.6 (34.9-160.4)	73.6 (28.5-147.1)

Data are presented as medians (minimum to maximum). ^a^: P<0.005 T2D vs C; ^b^ P<0.01 T2D vs GD; ^c^ P<0.05 T2D vs C; ^d^: P<0.0001 T2D vs GD; ^e^: P<0.0001 T2D vs C.

Moreover, VCB levels of SHBG and DHEAS were lower in T2D compared to DG (**Table 2**, P< 0.0001 and P<0.01, respectively) and C (**Table 2**, P<0.05 and P<0.0001, respectively). However, testosterone, androstenedione, the free androgen index, estradiol, estrone, and estriol levels were similar among the VCB of the three groups.

No significant correlation was found between VCB levels of AMH, DHEAS, testosterone, estrogens, and SHBG and their corresponding maternal hormonal plasma levels, as reported in **Supplementary Table S1**.

### Metabolic profiles in VCB samples

VCB insulin levels, HOMA-IR index, and IGF-1 were significantly higher in pregnancies with T2D compared to C and GD groups (**Table 3**, P<0.001, P<0.05 and P<0.05, respectively). Furthermore, VCB adiponectin levels were lower in the T2D group than in the C group (P < 0.05).

**Table 3:** Metabolic profile in plasma of venous cord blood of T2D, GD, and control pregnancies.

Venous blood cord levels	T2D	GD	Control
n	24	26	25
Glucose (mg/dl)	39.2 (10.0-70)	36.5 (31.7-74.9)	40.0 (32.0-48.0)
Insulin (μIU/ml)	8.4 (1.5-86.0)^a^	3.4 (0.6-11.9)^b^	3.8 (0.2-26.1)
HOMA -IR	0.7 (0.1-11.6) ^c,d^	0.3 (0.06-0.8)	0.4 (0.02-2.9)
IGF-1 (ng/ml)	117.0 (74. 7-172.6)^c,e^	74.7 (33.6-127.4)	87.5 (38.5-122.2)
IGFBP-1 (ng/ml)	39.9 (12.0-214.2)	42.2 (8.0-216.1)	53.2 (7.3-230.8)
Adiponectin (μg/ml)	38.4 (9.6-52.4)^c^	43.7 (23.9-69.7)	46.4 (32.7-63.5)

The data are presented as medians (minimum to maximum). Symbols, ^a^P< 0.001 for T2D vs. C, and ^b^P< 0.001 for GD vs. C. ^c^: P<0.05 T2D vs C, ^d^ p<0.001 for T2D vs GD; ^e^ p<0.0001 for T2D vs GD.

No significant correlation was found between VCB insulin, APN, and IGF-1 levels and their corresponding levels in maternal plasma, as reported in **Supplementary Table S1**.

### Hormonal profile in pregnancies with AGA and LGA offspring

In the AGA subgroup, the differences in VCB hormonal and metabolic profiles between pregnancies with T2D, and those with GD, and control pregnancies remained significant, consistent with the findings in the entire cohort. (**Supplementary Table S2**). The number of cases born LGA in the C group was too low for analysis due to insufficient statistical power.

## Discussion

This study investigated AMH, sex steroid levels, and metabolic profiles in VCB from 75 pregnancies with T2D, GD, and normal glucose levels during pregnancy. This study investigated AMH, sex steroid levels, and metabolic profiles in VCB from 75 pregnancies with T2D, GD, and normal glucose levels during pregnancy. To our knowledge, this is the first study to explore AMH and sex steroid levels and their relationship with markers of insulin sensitivity in cord blood in pregnancies affected by hyperglycemia, which previously had been studied during pregnancy but not at delivery in cord blood (13, 17, 18). Our data show that higher AMH levels and lower SHBG, DHEAS, and APN levels, along with higher insulin levels and HOMA-IR scores, in cord blood of pregnancies with T2D, suggests that this type of pregestational diabetes may be associated with changes in hormones that regulate ovarian development and metabolic profile in the intrauterine environment of the offspring.

The higher AMH levels in VCB that we observed in T2D pregnancies may be related to changes in the placental secretion of AMH. The placenta and fetal membranes express AMH and AMH receptor mRNAs at term (19). Further support that elevated VCB AMH levels originate from placental sources is based on several facts: in our previous study of this same cohort, maternal circulating AMH levels were not elevated during pregnancy in women with T2D (13). Additionally, VCB AMH levels did not show a correlation with maternal AMH levels (see **Supplementary Table S2**). We propose that the insulin-resistant and hyperinsulinemic environment observed in pregnancies affected by type 2 diabetes (T2D) may have contributed to increased secretion of AMH by the placenta. Insulin resistance plays a role in elevated AMH in PCOS (20–22). Our data showing elevated AMH in VCB of T2D pregnancies suggests that pregnancies affected by T2D have a similar profile to that observed in cord blood of pregnancies with PCOS, which also have elevated AMH and normal testosterone levels (17, 22–24). Elevated testosterone levels were not observed in VCB of pregnancies with T2D, as we have previously shown in the plasma of mothers with T2D during the second half of pregnancy (12, 13). Furthermore, our study did not find a correlation between testosterone levels in VCB and maternal plasma levels. This finding is consistent with observations in pregnant women with PCOS, where maternal hyperandrogenism is observed during pregnancy but not in the newborn (25). Placental aromatase and elevated SHBG levels have been postulated to reduce fetal exposure to maternal androgens (26–28), and could be the factors explaining the differences in VCB and maternal testosterone levels in pregnancies with T2D.

DHEAS levels were lower in T2D than in the VCB of the GD and C groups. DHEAS is synthesized mainly by the placenta and the fetal zone (FZ) of the adrenal gland (29, 30). At the time of birth, the adrenal gland undergoes significant reorganization, which is characterized by the involution of the FZ. This event leads to a sharp decrease in DHEAS levels, restored during adrenarche (29, 31). Lower levels of DHEAS observed in VCB of T2D may indicate a dysfunction in placental steroidogenesis or early FZ dysfunction induced by T2D.

In the current study, T2D was associated with higher VCB levels of insulin, a higher HOMA-IR index, lower SHBG and adiponectin levels. The latter suggests that insulin resistance is present at the time of delivery in pregnancies with T2D, and that this type of diabetes disrupts the intrauterine environment where the female fetus develops. These findings are part of the response to counteract maternal higher blood glucose levels and increased nutrient influx to the fetuses during pregnancies complicated by T2D (32, 33).

The clinical phenotype and VCB hormonal profile were different in pregnancies with T2D and GD, which may be explained by the fact maternal diabetes exposure occurs earlier in T2D than in GD. During pregnancies affected by T2D, the fetal-placental unit is exposed to maternal hyperglycemia and hyperinsulinemia in the early period of organ development. In contrast, in pregnancies with GD, hyperglycemia appears at approximately 24-28 weeks of gestation, at a time when the placenta and fetal ovary are less sensitive to maternal hyperglycemia and fetal hyperinsulinemia (34). The main strength of this research is the follow-up of the mother-offspring pairs begun in the second trimester, with a precise diagnosis of the type of diabetes. Another strength of the research is that the blood collection was obtained from the VCB, which offers a reliable representation of the intrauterine environment (10, 35). Some limitations must be addressed. This study lacked a clinical assessment regarding adipose tissue deposition and a lack of follow-up on the offspring during mini-puberty. Another limitation of the study was that participants were recruited at a single center, which may introduce some bias. Multicenter studies are necessary to confirm these findings.

In summary, the hormonal profile in VCB of T2D pregnancies shows increased levels of AMH, insulin, IGF-1, and lower APN levels. These abnormalities were not observed in GD pregnancies. The fact that VCB obtained from AGA T2D had elevated AMH levels and a hormonal profile indicative of insulin resistance indicates that T2D was the primary factor explaining the results, rather than birth weight. Despite the hyperandrogenemia observed in T2D, androgens are not elevated in VCB. In conclusion, T2D induces changes in the intrauterine environment, characterized by increased insulin, IGF-1, and AMH levels and decreased adiponectin levels in VCB samples. Future studies should evaluate whether the findings we observed at birth could affect reproductive function later in postnatal life in girls.

## Data Availability

The raw data supporting the conclusions of this article will be made available by the authors, without undue reservation.
