# Supplementary material for: Anti-Müllerian hormone, sex steroids, and metabolic profile in cord blood of pregnancies with type 2 diabetes and gestational diabetes
Source: Front Endocrinol (Lausanne). 2025 Jul 28;16:1589541. doi: 10.3389/fendo.2025.1589541 (PMC12336012; doi:10.3389/fendo.2025.1589541)
Supplement: Supplementary file 1 [file Table1.docx]

**Supplementary Table 1:** Hormonal and metabolic profile in plasma of venous cord blood of pregnancies that resulted in offspring with appropriate weight for their gestational age.

| **Hormonal Parameters in VCB** | **T2D** | **GD** | **Control** |
| --- | --- | --- | --- |
| **n** | 12 | 14 | 19 |
| **AMH (ng/ml)** | 2.0 (0.1-3.6)^a,b^ | 0.7 (0.1-1.2) | 0.2 (0.1-1.2) |
| **SHBG (nmol/l)** | 169.7 (132-243) ^b^ | 195.3 (172.7-312.4) | 188.9 (81-232) |
| **Testosterone (ng/ml)** | 2.0 (1.0-3.65) | 1.2 (0.7-6.9) | 1.7 (0.2-3.6) |
| **Androstenedione (ng/ml)** | 28.9 (19.3 - 40.0) | 20.1 (14.2-40) | 24.4 (5.6-35.5) |
| **DHEAS (μg/dl)** | 172.1 (133.6-243.8)^c^ | 198.3 (75.5-257.6) | 216.7 (113.6-366.7) |
| **Free Androgen Index (%)** | 4.4(1.9–7.1) | 2.2 (1.5-7.7) | 3.4 (0.3-6.4) |
| **Estradiol (ng/ml)** | 11.0 (7.4-23.9) | 11.1 (5.2-145.5) | 9.6 (4.7-26.1) |
| **Estrone (ng/ml)** | 8744.5 (5013 - 20593) | 12916.5 (4678-34688) | 11443 (2101-36014) |
| **Estriol (ng/ml)** | 65.9 (32-109.2) | 58.1 (34.9-160.4) | 68.7 (28.5-119.9) |
| **Metabolic Parameters** |  |  |  |
| **Glucose (mg/dl)** | 38.3 (17.8 -49) | 38.9 (31.7-70) | 40.0 (32.0-45.1) |
| **Insulin (μIU/ml)** | 7.0 (1.5-23.3)^a,b^ | 2.9 (0.6-11.9) | 3.8 (0.2-26.1) |
| **HOMA -IR** | 0.7 (0.1-2.8) ^c^ | 0.2 (0.06-0.8) | 0.4 (0.02-2.9) |
| **IGF-1 (ng/ml)** | 116.1 (74.7-172.6)^a,d^ | 62.8 (33.6-127.4) | 84.5 (38.5-122.2) |
| **IGFBP-1 (ng/ml)** | 42.9 (12.9 -110.6) | 44.8 (8.0-216.1) | 50.2 (7.3-230.8) |
| **Adiponectin (μg/ml)** | 29.0 (9.6-52.4)^a,c^ | 52.2 (23.9-66.5) | 46.5 (32.7-63.5) |

**Supplementary table 1**: Data are presented as medians (minimum to maximum). a: P<0.001 T2D vs C. ;b P<0.05 T2D vs GD; c P<0.05 T2D vs C; d p<0.001 for T2D vs GD
