# Supplementary material for: Anti-Müllerian hormone, sex steroids, and metabolic profile in cord blood of pregnancies with type 2 diabetes and gestational diabetes
Source: Front Endocrinol (Lausanne). 2025 Jul 28;16:1589541. doi: 10.3389/fendo.2025.1589541 (PMC12336012; doi:10.3389/fendo.2025.1589541)
Supplement: Supplementary file 2 [file Table2.docx]

| **Correlation of hormones in maternal and umbilical cord samples** | **r** | **p** |
| --- | --- | --- |
| **AMH (ng/ml)** | -0.129 | 0.269 |
| **SHBG (nmol/l)** | -0.180 | 0.121 |
| **Testosterone (ng/ml)** | -0.041 | 0.730 |
| **Androstenedione (ng/ml)** | -0.067 | 0.568 |
| **DHEAS (mg/dl)** | -0.002 | 0.986 |
| **Adiponectin (mg/ml)** | 0.195 | 0.094 |
| **Insulin (mIU/ml)** | -0.084 | 0.476 |
| **IGF-1 (ng/ml)** | 0.207 | 0.075 |
| **IGFBP-1 (ng/ml)** | 0.0001 | 0.997 |

**Supplementary Table 2**: Pearson’s two-tailed test compared maternal and venous cord plasma hormone levels in pregnancies with T2D and GD, with a significance level of p < 0.05.
